# Design and Evaluation of a Novel Anti-microbial Peptide from Cathelicidin-2: Selectively Active Against *Acinetobacter baumannii*

**DOI:** 10.5812/ijpr-141920

**Published:** 2023-12-12

**Authors:** Fariba Fathi, Maryam Ghobeh, Farshad H Shirazi, Maryam Tabarzad

**Affiliations:** 1 Department of Biology, Science and Research Branch, Islamic Azad University, Tehran, Iran; 2 Department of Toxicology and Pharmacology, School of Pharmacy, Shahid Beheshti University of Medical Sciences, Tehran, Iran; 3 Pharmaceutical Sciences Research Center, Shahid Beheshti University of Medical Sciences, Tehran, Iran; 4 Protein Technology Research Center, Shahid Beheshti University of Medical Sciences, Tehran, Iran

**Keywords:** Anti-microbial Peptides, Design, Hemolytic Activity, *Acinetobacter baumannii*, Anti-fungal

## Abstract

**Background:**

Infections caused by pathogenic microorganisms have increased the need for hospital care and have thus represented a public health problem and a significant financial burden. Classical treatments consisting of traditional antibiotics face several challenges today. Anti-microbial peptides (AMPs) are a conserved characteristic of the innate immune response among different animal species to defend against pathogenic microorganisms.

**Objectives:**

In this study, a new peptide sequence (mCHTL131-140) was designed using the in silico approach.

**Methods:**

Cathelicidin-2 (UniprotID: Q2IAL7) was used as a potential antimicrobial protein, and a novel 10 - 12 amino acids sequence AMP was designed using bioinformatics tools and the AMP databases. Then, the anti-bacterial, anti-biofilm, and anti-fungal properties of the peptide, as well as its hemolytic activity and cytotoxicity towards human fibroblast (HDF) cells, were investigated in vitro.

**Results:**

Online bioinformatics tools indicated that the peptide sequence could have anti-bacterial, anti-viral, anti-fungal, and anti-biofilm properties with little hemolytic properties. The experimental tests confirmed that mCHTL131-140 exhibited the best anti-bacterial properties against *Acinetobacter baumannii* and had fair anti-fungal properties. Besides, it did not cause red blood cell lysis and showed no cytotoxicity towards HDF cells.

**Conclusions:**

In general, the designed peptide can be considered a promising AMP to control hospital-acquired infections by *A. baumannii*.

## 1. Background

The speedy appearance and dissemination of bacteria strains resistant to antibiotics have coincided with the ongoing use of antibiotics. According to predicting statistical models, 4.95 million deaths annually are caused by resistant bacteria, with at least 1.27 million deaths tied directly to antimicrobial resistance (1, 2). The lack of efficient treatments for infections brought on by these organisms significantly impacts the economy and well-being (3). Consequently, it is critical to create novel agents to tackle antibiotic resistance. One possible alternative to antibiotics for dealing with these problems is antimicrobial peptides (AMPs) (4-6).

Antimicrobial peptides are small bioactive compounds, biosynthesized by all organisms as vital parts of their innate immune system. They serve as the primary line of defense against microbial threats in eukaryotes or as a competitive strategy to limit other microorganisms' growth in prokaryotes (7-9). Numerous biological roles are associated with the antimicrobial activity of AMPs, including anti-inflammatory, adaptive immunity, chemotaxis, pro-inflammatory, and endotoxin neutralization, which are only a few of the immunomodulatory properties that AMPs can exhibit. Some AMPs are recognized to have anti-biofilm, wound healing, and angiogenesis actions (10).

Although these characteristics appear as promising features through drug development, some disadvantages have been pinpointed for AMP-based therapies, including chemical and physical instability, proteolytic degradation, short half-life and rapid elimination, slow tissue penetration, toxicity toward healthy human cells, and cell specificity. Based on that, an increasing number of computational strategies are underway, aiming to overcome these obstacles towards designing potent AMP sequences (11).

Recently, using bioinformatics tools in the design and development of novel AMPs has been widely studied (4, 12, 13). For example, a novel AMP (RFGRFLRKILRFLKK) was designed based on the sequence of chicken cathelicidin-2 using in silico methods considering the physicochemical features of active AMPs, which was found to be more effective and less toxic than the original template (14).

## 2. Objectives

In fact, free online web servers have been developed to forecast and identify new synthetic antimicrobial peptides. This research aimed to design a multifunctional antimicrobial peptide with anti-biofilm, anti-inflammatory, and wound-healing properties and minimum cytotoxicity, utilizing an in silico approach. Here, the primary study toward achieving an active AMP is reported, focusing on the design and evaluation of the anti-microbial activities of the newly designed peptide.

## 3. Methods

### 3.1. In silico Design of a Novel Peptide

A peptide was designed by using bioinformatics tools. Briefly, the initial stage involved finding a template sequence using the UniProt database (15). The template protein was determined to be cathelicidin-2 (UniProt ID: Q2IAL7). In the second stage, the cathelicidin-2 sequence was scanned using the following online predictive tools to identify a region with the requisite bioactive characteristics; the URLs of all the applied web servers were presented in Table 1. 

**Table 1.** The List of Online Tools Applied to Peptide Design

Name	Description	Link
**UniProt**	a comprehensive source of information on protein sequences and annotations	https://www.uniprot.org/
**CAMP**_**R4**_	Database of antimicrobial peptides and proteins	http://www.camp.bicnirrh.res.in/index.ph
**AntiFungal Webserver**	A prediction server for antifungal peptide	https://www.chemoinfolab.com/antifungal/query/
**dPABBs**	A tool for the prediction and design of anti-biofilm peptides	https://abopenlab.csir.res.in/abp/antibiofilm/index.php
**PreAIP**	An accurate predictor of anti-inflammatory peptides	http://kurata14.bio.kyutech.ac.jp/PreAIP/
**AIPpred**	A tool for the prediction of anti-inflammatory activity of peptides	http://thegleelab.org/AIPpred/index.htmL
**CellPPD**	A tool for the prediction and design of efficient cell-penetrating peptides	https://webs.iiitd.edu.in/raghava/cellppd/index.htmL
**HemoPI**	A tool for the prediction of hemolytic activity of peptides	https://webs.iiitd.edu.in/raghava/hemopi/design.php
**Meta-iAVP**	A tool for the prediction of antiviral peptides	http://codes.bio/meta-iavp/
**ExPASy tool, ProtParam**	A tool for the computation of various physical and chemical parameters	https://web.expasy.org/protparam/
**ToxinPred**	A tool for the prediction and design of toxic/non-toxic peptides	https://webs.iiitd.edu.in/raghava/toxinpred/
**PEP-FOLD3**	A web server for predicting peptide structures from amino acid sequences	https://bioserv.rpbs.univ-paris diderot.fr/services/PEP-FOLD3/
**EPIPOX**	A tool for predicting antigenic peptides	http://imed.med.ucm.es/Tools/antigenic.pl
**HeliQuest**	A tool for the calculation of helix properties	https://heliquest.ipmc.cnrs.fr/cgi-bin/ComputParams.py

The selected protein sequence was scanned by the CAMP_R4_ web service, which identified antimicrobial-active areas (16). After selecting the sequence with probable AMP property, the anti-biofilm property of the sequence was checked using the dPABBs online server (17). In the next step, the Meta-iAVP online server (18) was used to check for the antiviral property of the target sequence. This server is a predictor based on the sequence that reports the anti-viral property with numbers between 0 and 1. As in the previous cases, the closer this number is to 1, the more likely for the sequence to be an anti-virus.

Antifungal, hemolytic, antigenic, and toxic characteristics of the peptide sequence were also checked using the relevant servers. Peptide physical and chemical properties, such as molecular weight, isoelectric point, net positive charge, Bowman's index, and hydrophobicity, were calculated using the ExPASy tool, ProtParam, and ADP3 websites (19). Also, the peptide's three-dimensional structure and helical wheel were obtained using the PEP-FOLD (20) and HeliQuest websites (21), respectively.

### 3.2. Peptide Synthesis

GenScript (China) was commissioned to carry out the chemical synthesis of the peptide using the Fmoc-based solid-phase technology. The company certificate confirmed the peptide identity and purity after purification by high-performance liquid chromatography (HPLC), and the peptide molecular weight was verified through LC-Mass spectroscopy (6410 QQQ, Agilent, USA, conducted by GenScript). The peptide was received in lyophilized form and was kept at -80°C until use. Afterward, the peptide solution was prepared with sterile water for injection at a concentration of 10 mg.mL^-1^ and kept at -20°C.

### 3.3. Microorganism Strains

The following microorganisms were used in this study: methicillin-resistant *Staphylococcus aureus* (ATCC33591), *S. aureus* (ATCC25923), *Pseudomonas aeruginosa* (ATCC27853), *Klebsiella pneumonia* (ATCC10031), *Enterobacter aerogenes* (ATCC10687), *Enterococcus faecium* (ATCC10836), *Escherichia coli* (ATCC25922), and a previously collected and confirmed clinical sample of *Acinetobacter baumannii. Candida albicans* (ATCC10231) and *Aspergillus flavus* (ATCC16404) were used as two samples of pathogenic fungi.

### 3.4. Phylogenic Analysis

The peptide sequence was analyzed using the ADP3 database (19) to determine how similar it was to the sequences of known antimicrobial peptides. Then, the top 10 sequences resembling the newly discovered peptide with the most similarity were taken. A phylogenetic tree was then constructed after they were realigned using the Clustal Omega online server from the EMBL-EBI website (22).

### 3.5. Evaluation of Antibacterial Activity

Antibacterial activity was evaluated using microdilution (23, 24). Through the microdilution approach, an initial 100 µL of the Mueller-Hinton broth medium (MHB) was added into each well of a 96-well microplate. The initial concentration of the peptide was 1 mg.mL^-1^. Serial dilutions of the positive control, polymyxin, and peptide (0.48 - 500 µg.mL^-1^) were produced in separate wells in triplicate. Then, 10 µL of a 1:20 dilution of 0.5 McFarland suspension (1× 10^8^ CFU.mL^-1^), containing 5 × 10^6^ CFU.mL^-1^, was added to each well. The plates were incubated for 24 hours at 35 ± 2°C before the bacterial growth was measured by reading the absorbance at 630 nm.

### 3.6. Evaluation of Antifungal Activity

The Clinical & Laboratory Standards Institute (CLSI) international method (25, 26) was used to determine the minimum inhibitory concentration (MIC) of the fungi. Briefly, a suspension of fungal cells was prepared with a concentration of 2.5 × 10^3^ cells.mL^-1^. Next, 100 µL of the cell suspension was introduced into all wells of a 96-well microplate. Then, 50 µL of the peptide sample with different concentrations (ranging from 1000 to 7.8 µg.mL^-1^) was added to each well. Nystatin was used as a control sample. The positive control contained the fungal suspension and the RPMI 1640 medium, and the negative control contained the peptide sample and medium. Finally, after 20 hours of incubation at 35°C, the absorbance was recorded at 600 nm.

### 3.7. Evaluation of Anti-biofilm Activity

The approach described by Haney et al. (27) was modified to measure the anti-biofilm activity. Briefly, the inhibitory and eradication properties of the biofilm were evaluated to investigate the anti-biofilm activity of the designed peptide. *Pseudomonas aeruginosa, A. baumannii*, and *S. aureus* were selected as the candidate bacteria forming the biofilm. A cell suspension with OD600 = 0.01 was prepared from a 24-hour bacterial culture. Different peptide concentrations were prepared by serial dilution. Each well received 10 µL of the peptide serial dilution (62.5 - 1000 µg.mL^-1^), and then 90 µL of the cell suspension was added to the wells. The sample without the peptide (culture medium and bacteria) was considered as the growth control, and the wells including just the medium (neither peptide nor bacteria) were considered as the sterility control. The microplate was incubated for 20 hours at 37°C. The absorbance was measured at 600 nm to ascertain the rate of bacterial growth. After washing the wells, 105 µL of crystal violet (CV) 0.1% was added to each well. It was incubated for 30 minutes before the excess dye was discarded. Subsequently, 110 µL of 70% ethanol was added to each well, and after 30 minutes of incubation at ambient temperature, the absorbance was read at 570 nm, and the percentage of bacterial growth and biofilm formation was calculated using Equations respectively:


Equation 1. $$\%Bacterial\;growth=100\times \frac{\left({sampleOD}_{600}-{SCOD}_{600}\right)}{\left({GCOD}_{600}-{SCOD}_{600}\right)}$$

Equation 2. $$\%Biofilm\;formation=100\times \frac{\left({sampleA}_{570}-{SCA}_{570}\right)}{\left({GCA}_{570}-{SCA}_{570}\right)}$$

Where sampleOD_600_ is the optical density (OD) of the sample at 600 nm, and sampleA_570_ is the absorbance of the sample at 570 nm. Moreover, SC is the sterility control, and GC is the growth control.

A cell suspension with an OD_600_ of 0.01 was prepared for the biofilm eradication assay. Briefly, 100 µL of this cell suspension was added to each well and incubated at 37°C for 24 hours. After discarding the medium and washing the wells, 180 µL and 20 µL of the serially diluted peptide were added to each well. Then, 10 µL of 5% TTC (triphenyltetrazolium) was added and incubated for 24 hours at room temperature. The contents of each well were discarded, and 205 µL of DMSO (dimethyl sulfoxide) was added to each well after washing. Finally, it was kept at room temperature for 30 minutes before the absorbance was measured at 500 nm. Equation was used to calculate the percentage of biofilm metabolism.


Equation 3. $$\%Biofilm\;metabolism=100\times \frac{\left({sampleA}_{500}-{SCA}_{500}\right)}{\left({GCA}_{500}-{SCA}_{500}\right)}$$

### 3.8. Evaluation of Hemolytic Activity

The hemolytic activity of the peptide was measured according to a previously used method (28). First, 5 mL of fresh blood was centrifuged at 4000 g for 5 minutes, the supernatant was discarded, and the blood cell sediment was diluted to a final volume of 20 mL using PBS (phosphate-buffered saline). In PBS, the peptide sample was serially diluted (1 to 0.0312 mg.mL^-1)^. In a micro tube, 10 µL of the peptide solution was combined with 190 µL of the cell suspension (The peptide concentration in the final mixture was 50 µg.mL^-1^ to 1.5 µg.mL^-1^). The tubes were then kept for 30 minutes at 37°C and centrifuged for 5 minutes at 4000g. The absorbance was measured at 567 nm after 100 mL of the supernatant was diluted in 1 mL of the PBS buffer. The release of hemoglobin is indicative of red blood cell membrane breakdown. In the buffer containing PBS and 0.2% Triton X-100, a negative control without hemolysis and a positive control with 100% hemolysis were determined, respectively. The proportion of hemolysis was estimated using Equation


Equation 4. $$Hemolysis\left(\%\right)=\frac{A_{S}-A_{0}}{A_{100}-A_{0}}\times 100$$

where A_s_ is the absorbance at 567 nm of the sample, A_100_ is the absorbance of completely lysed red blood cells in 0.2% Triton X-100, and A_0_ is the absorbance without hemolysis.

### 3.9. Cytotoxic Assessment of mCHTL (131-140)

The 3-(4,5-dimethylthiazol-2-yl)-2,5-diphenyltetrazolium bromide (MTT) assay (29) was used to evaluate the toxicity of mCHTL (131-140) on human dermal fibroblast )HDF) cell line. In brief, HDF cells were cultured in DMEM (Gibco, low-glucose Dulbecco's modified eagle medium) with 10% (v/v) fetal bovine serum (DNA Biotech, FBS) and antibiotics (100 U/mL penicillin and 100 U/mL streptomycin) at 37°C in a 5% CO_2_ environment. Then, 5×10^3^ cells per well were seeded to 96-well microplates containing basic DMEM without serum (90 µL), and the cells were cultured for 24 hours until they adhered to the wall. Afterward, each well received 10 µL of the serial dilution of peptide (from 1,000 to 7.8 µg.mL^-1^) and underwent a 24-hour incubation. PBS served as the negative sample control. The microplate was then incubated at 37°C with 5% CO_2_ for 4 hours before 10 µL of the filtered MTT (3-(4,5-dimethylthiazol-2-yl)-2,5-diphenyltetrazolium bromide) solution was introduced to each well. After discarding the supernatant, the samples were agitated with 20 µL of DMSO (dimethyl sulfoxide) for 30 minutes, and their absorbance at 570 nm was measured (Garni Medical Engineering Co., Iran).

### 3.10. Peptide Structural Study by Circular Dichroism

For peptide structure analysis, it was dissolved in 1x PBS solution (1000 µg.mL^-1^), and its structure was then analyzed by circular dichroism (CD) spectropolarimeter (J-715, JASCO, Japan).

### 3.11. Statistical Analysis

The results were presented as mean ± standard deviation (SD) from 3 independent tests. Comparison of means between different groups of samples was performed using a two-way analysis of variance (ANOVA). All the statistical analyses were performed using GraphPad Prism v. 9.0, and a P-value < 0.05 was considered significant.

## 4. Results

### 4.1. Novel AMP [mCHTL (131-140)] Designed by Bioinformatics Tools

The amino acid sequence of cathelicidin-2, a chicken protein that can bind to lipopolysaccharides from bacteria, was selected as the primary template. The antibacterial capabilities of this protein against both Gram-positive and Gram-negative bacteria have been reported. Moreover, it has hemolytic characteristics through in vitro experiments and is expected to have a function in the innate immune system (30).

Therefore, cathelicidin-2 was deemed a suitable template for common antibacterial peptides' length ranging between 10 to 60 residues (31). Since the cost of peptide synthesis is one of the main obstacles to the introduction of these drug candidates into the pharmaceutical industry, the main focus was on the design of short-length peptides. Therefore, the initial length of 10 amino acids was considered in this study for designing a novel AMP.

A 10-residue sequence was selected after scanning the template protein sequence using the CAMPR4 server. The threshold value of 0.8 was set for all algorithms to increase accuracy and achieve better performance. The selected sequence (RFLRKIRRFR) was located at positions 131-140 of the cathelicidin-2 protein sequence. The results of investigating the antimicrobial properties of this sequence using support vector machine (SVM), random forest (RF), and artificial neural network (ANN) algorithms are reported in Table 2. 

**Table 2.** The Characteristics of the Template Protein and the Selected Region as a Putative AMP

UniProt ID	Protein Name	Protein Sequence	The Selected Sequence	Position	AMP (SVM)	AMP (RF)	AMP (ANN)
**Q2IAL7 (CTHL2_CHICK)**	Cathelicidin-2	MLSCWVLLLALLGGVCALPAPLSYPQALIQAVDSYNQRPEVQNAFRLLSADPEPGP GVDLSTLRALNFTIMETECTPSARLPVDDCDFKENGVIRDCSGPVSVLQDTPEINLRC RDASSDPVLVQRGRFGRFLRKIRRFRPKVTITIQGSARFG	RFLRKIRRFR	131-140	1	0.68	0.98

Abbreviations: AMP, anti-microbial peptide; ANN, artificial neural network; RF, random forest; SVM, support vector machine.

Considering that the score reported by the RF algorithm was 0.68 (less than the threshold number), some changes were made to increase the probability of antimicrobial activity. The specifications of the new sequence are reported in Table 3. By replacing 2 phenylalanine (F) amino acids in the second and ninth positions of the selected sequence with tryptophan (W) and then adding 2 extra lysines (K) to the initial AMP sequence (one at the end of the sequence and the other one after the first amino acid), a 12-residue peptide with an enhanced antimicrobial potential was created (RKWLRKIRRWRK) and named as mCHTL (131-140).

**Table 3.** Comparison of the Characteristics of Both Initial and Modified Sequences

	Initial Sequence (RFLRKIRRFR)	New Sequence (RKWLRKIRRWRK)
**AMP (SVM)**	0.999	0.95
**AMP (RF)**	0.68	1.00
**AMP (ANN)**	0.98	0.99
**Antibiofilm (SVM)**	0.19 (anti-biofilm inactive)	1.32 (anti-biofilm)
**Antibiofilm (WEKA)**	0.81 (anti-biofilm)	1.00 (anti-biofilm)
**Antiviral**	1 (anti-viral)	1.00 (anti-viral)
**Anti-fungal**	-	78.8
**Anti-inflammatory**	0.576 (high confidence AIP)	0.652 (high confidence AIP)
**Hemolytic potency**	0.43	0.35
**Toxic peptide**	-1.21 (non-toxic)	-0.85 (non-toxic)
**Total hydrophobic ratio**	40%	33%
**The molecular weight**	1447.799	1782.211
**The total net charge**	+6	+8
**PI**	12.60	12.61
**Hydrophobicity (H)**	0.104	-0.002
**Hydrophobic moment (µH)**	0.727	0.803
**Boman indexkcal/mol**	6.43	6.39
**GRAVY**	-1.25	-2.308
**Similarity percentage**	46.15%	50%
**Similar peptide (APD ID and sequence of peptide)**	AP00008 (RLCRIVVIRVCR)	AP02856 (WWWLRKIW)

Abbreviations: AMP, anti-microbial peptide; SVM, support vector machine; RF, random forest; ANN, artificial neural network.

The mCHTL131-140 was introduced with a high probability of possessing anti-biofilm properties. Moreover, a minor hemolytic property (~35%) of mCHTL131-140 was observed.

The Meta-iAVP server dedicated a score of 1 to both sequences (the initial and modified ones). It means that both were 100% likely to have antiviral functionality. The sequence's antifungal index was projected by an antifungal webserver to be 78.8%, and it was 99% probable to exhibit antifungal activities against *C. albicans*.

The physical and chemical properties of the peptide were calculated using HeliQuet and ExPASY servers (Table 3). As demonstrated, the net positive charge of mCHTL131-140 was +8. The total hydrophobic ratio was 33%, and hydrophobicity was negative (- 0.002 H). In addition, the peptide had a Boman index of 6.39 kcal.mol-1, the grand average of the hydropathicity index (GRAVY) was -2.308, and the hydrophobic moment was 0.803 µH.

**Figure 1.**
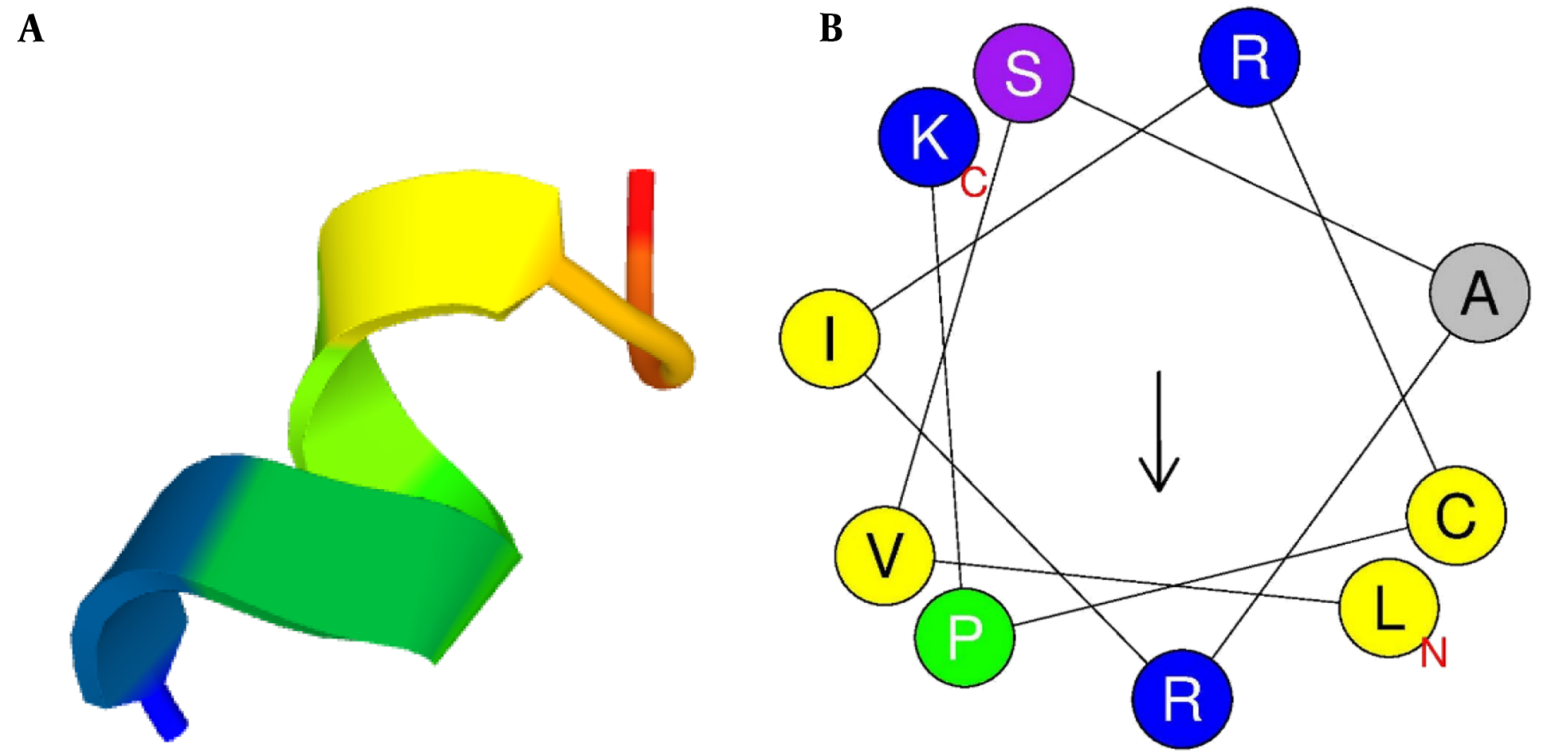
(A) Three-dimensional structure of the peptide simulated by PEP-FOLD3; (B) Graphic design of the peptide's helical wheel drawn up by HeliQuest server. Yellow circles represent hydrophobic residues, and blue circles represent amino acids with positive charges. The arrow illustrates the direction of hydrophobic moment.

The three-dimensional structure of mCHTL131-140 was predicted using the PEP-FOLD3 server. It showed that the peptide would adopt an alpha-helix structure in vivo. Due to the significance of the helical wheel in the exploration of peptide hydrophobicity properties, the HeliQuest server was used to create the graphical diagram. It showed that the peptide had 2 different sides: a large cationic side with almost all cationic residues (R_1_, K_12_, R_5_, R_9_, K_2_, K_6_, and R_8_) and a small hydrophobic side (W_3_, W_10_, and L_4_) with only 1 basic amino acid, R11 (Figure 1). 

### 4.2. Comparing the Novel mCHTL131-140 with Previously Reported AMPs

**Figure 2.**
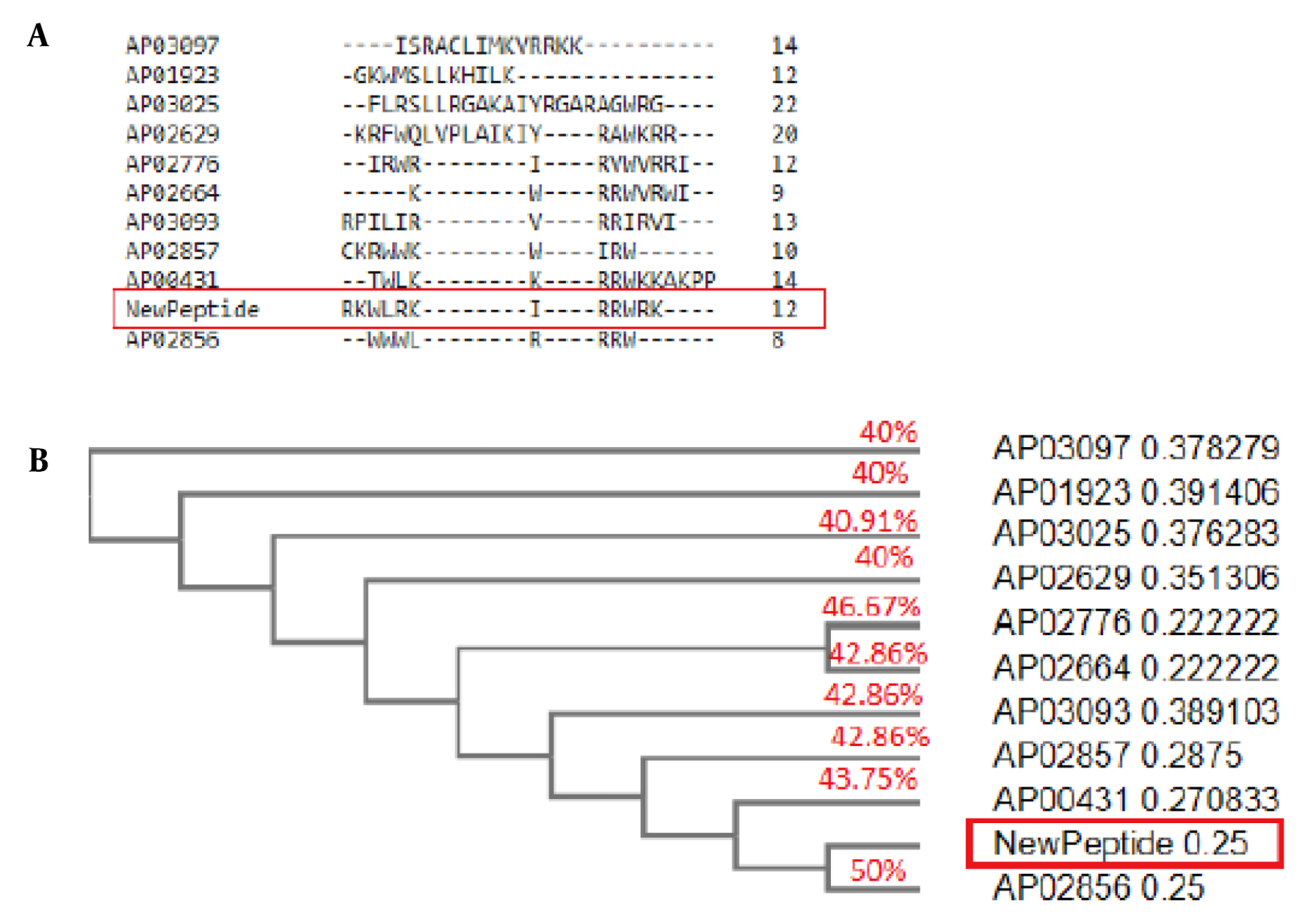
(A) BLAST results of 10 anti-microbial peptides (AMPs) sequences with the novel AMP. Peptides are designated by ADP ID. The new peptide is shown by "NewPeptide" in a red box; (B) Phylogenetic tree. As shown in the figure, the new peptide has the greatest similarity to Horine (AP02856), an artificial peptide designed based on temporin-SHf.

The mCHTL131-140 had the most homology with Horine (AP02856), an artificial peptide designed based on temporin-SHf. It is active against *E. faecium* V286-17, *S. aureus* USA300 LAC (MRSA), *K. pneumoniae* E406-17, *A. baumannii* B28-16, *P. aeruginosa* E411-17, and *E. coli E423-17* (32). The similarity of mCHTL131-140 to Horine was 50%. IDR-1010 (AP02776) is another peptide that was 46.67% similar to mCHTL (131-140). It is an Arg-rich peptide that can inhibit the growth of *S. aureus* and *P. aeruginosa* (33). The similarities of Cyclic L27-11 (AP00431), Pac-525 (AP02664), Inverso-CysHHC10 (AP02857), Peptide 19347_2 (AP03093), dicentracin-like peptide (AP03025), Halictine 2 (AP01923), Peptide 8361_2 (AP03097) and dCATH (AP02629) to mCHTL131-140 have also been demonstrated in Figure 2. It was anticipated that mCHTL131-140 would exhibit antibacterial properties against both gram-positive and -negative bacteria based on the activities reported for the most similar peptide sequences, along with the outcomes of bioinformatics analyses that predicted properties for this sequence.

### 4.3. Antibacterial Activity of mCHTL (131-140)

The microdilution method was implemented to determine the minimum inhibitory concentration (MIC) of the designed AMP. The results showed that mCHTL131-140 had the best performance in inhibiting the growth of *A. baumannii*. In the presence of *A. baumannii*, the MIC value of the peptide was 7.8 μg.mL^-1^, while polymyxin B did not show any inhibitory effect on this bacterium. The results of the antimicrobial activity evaluation are summarized in Table 4. 

**Table 4.** The Results of the Microdilution Test to Determine the MIC of the Peptide Against Different Bacteria

Bacteria	MIC (μg.mL^-1^)	
	Peptide	Polymyxin B
***Staphylococcus aureus***	≥ 250	≥ 0.97
***Pseudomonas aeruginosa***	≥ 62.5	≥ 1000
***Acinetobacter baumannii***	≥ 7.8	≥ 1000
***MRSA***	≥ 500	≥ 250
***Klebsiella pneumonia***	≥ 250	≥ 125
***Enterobacter aerrogenes***	≥ 500	≥ 1000
***Escherichia coli***	≥ 250	≥ 62.5
***Enterococcus faecium***	≥ 62.5	≥ 0.97

Abbreviation: MIC, minimum inhibitory concentration.

### 4.4. Antifungal Activity of mCHTL (131-140)

The lowest concentration of mCHTL131-140 required for inhibiting the growth of *C. albicans* was 62.5 μg.mL^-1^, while this value was 7.8 μg.mL^-1^ for nystatin as the positive control sample. The MIC of mCHTL131-140 on *A. flavus* was 31.25 μg.mL^-1^, whereas it was 3.9 μg.mL^-1^ for nystatin.

### 4.5. Hemolytic Activity of mCHTL (131-140)

The hemolytic activity of mCHTL131-140 was measured using Jang's method (13, 34). Based on this approach, after inserting the obtained absorption values in Equation (5), the percentage of hemolysis of blood cells was calculated. If the highest absorption value from the triton-X100 treated samples was considered as 100% hemolysis, the results showed that the values of red blood cell hemolysis percentage were near 0 after half an hour of peptide treatment in a concentration range of 1 to 0.03 mg.mL^-1^ (Table 5). The proposed peptide's lack of hemolytic activity is advantageous because many natural and even artificially synthesized AMPs have limited use due to their potent hemolytic properties.

**Table 5.** Hemolytic Activity of mCHTL 131-140

Concentration mg.mL^-1^	Ab (R1) 570 nm	Ab (R2) 570 nm	Ab (R3) 570 nm	% Hemolytic Activity, Mean ± SD
**1**	0.007	0.008	0.008	0.355731 ± 0.0395
**0.5**	0.007	0.007	0.007	0.250329 ± 0.0456
**0.25**	0.006	0.007	0.007	0.27668 ± 0.0395
**0.125**	0.000	0.000	0.001	0.184453 ± 0.114
**0.0625**	0.005	0.005	0.006	0.184453 ± 0.0604
**0.03125**	0.006	0.006	0.006	0.184453 ± 0.0913
**control + (Triton X100)**	1.98	2.53	2.53	91.04084 ± 7.918
**control – (PBS)**	0.008	0.008	0.008	0.250329 ± 0.0604

Abbreviations: SD, standard deviation; PBS, phosphate buffered saline

### 4.6. Anti-biofilm Activity of mCHTL (131-140)

The anti-biofilm activity of mCHTL131-140 was determined using the method presented by Haney et al. (27). By this method, both the inhibitory and the biofilm eradication activities of mCHTL131-140 were evaluated. Since *S. aureus* and *P. aeruginosa* are the most frequently isolated pathogens from chronic wounds, they were chosen primarily. In addition, *A. baumannii* was selected as another candidate for the biofilm test since the peptide showed the best antibacterial activity against it. The results of the antibacterial test were taken into account in order to select peptide concentrations. From the antibacterial test, it was found that the minimum concentration needed to inhibit the growth of *P. aeruginosa* was 62.5 μg.mL^-1^. In comparison, the minimum concentration against *S. aureus* was 250 μg.mL^-1^, and that against *A. baumannii* was 7.4 μg.mL^-1^. As a result, serial dilutions of the peptide with a minimum concentration of 62.5 µg.mL^-1^ and a maximum concentration of 1000 μg.mL^-1^ were selected.

**Figure 3.**
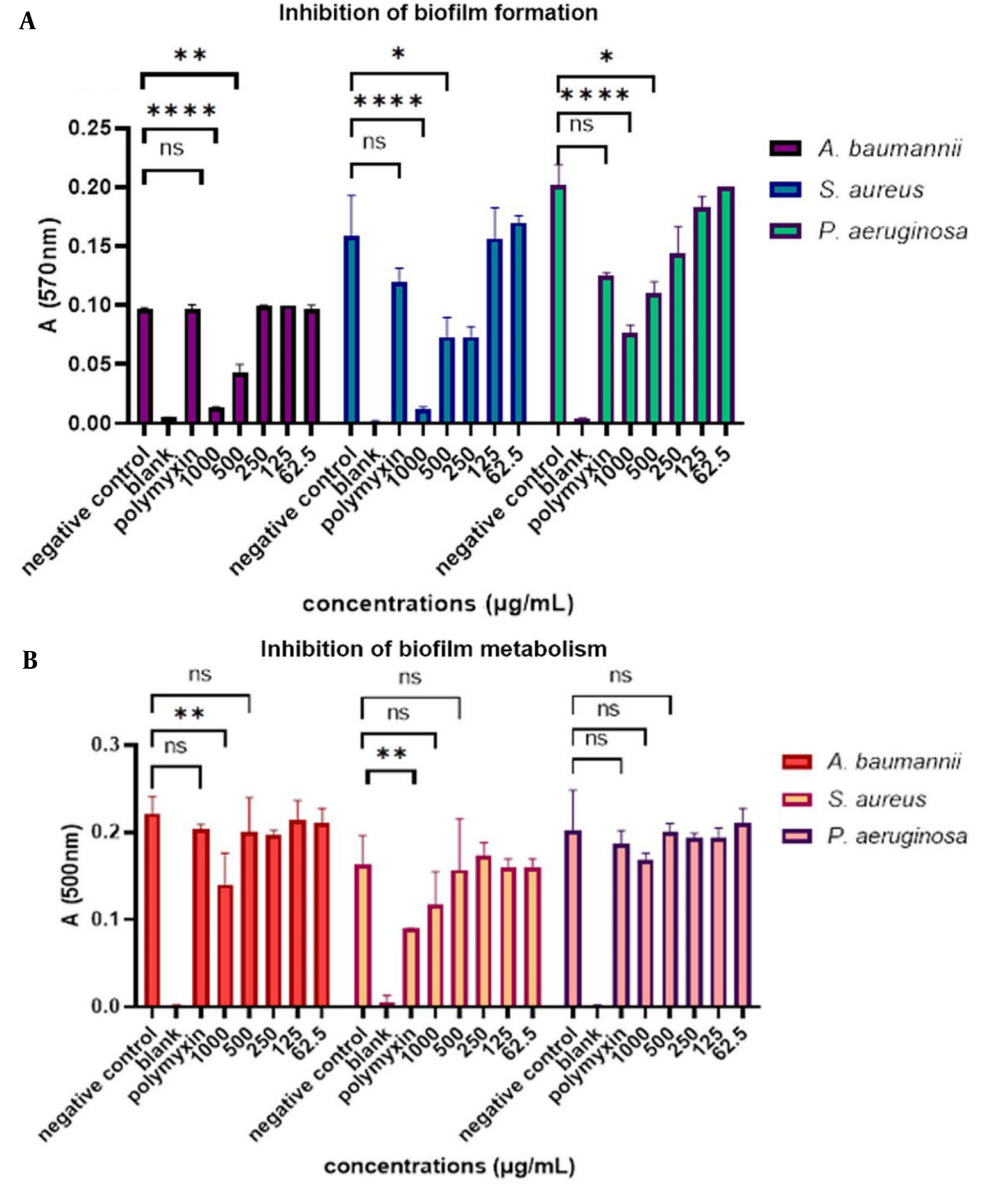
(A) Bar graph of the inhibitory effect of mCHTL131-140 on biofilm formation. As shown, it had the most significant inhibitory effect on *Staphylococcus aureus* biofilm, followed by *Acinetobacter baumannii*. It showed the lowest inhibitory effect on *Pseudomonas aeruginosa*; (B) Bar graph of the inhibitory effect of mCHTL131-140 on biofilm metabolism. As seen, it had a slight effect on inhibiting biofilm metabolism. A two-way analysis of variance was performed using GraphPad Prism v. 9.0. ns means the difference was non-significant, * P-value<0.05, ** P-value<0.01, **** P-value<0.0001.

The results showed that mCHTL131-140 at a concentration of 1000 µg.mL^-1^ had the best inhibitory effect on *S. aureus* biofilm and prevented biofilm formation by 93.3%. Besides, it had a lesser biofilm inhibition effect on *P. aeruginosa* as it could inhibit about 60% of biofilm formation at this concentration. The *A. baumannii* biofilm was inhibited by 85.5% in the presence of 1000 μg.mL^-1^ of the peptide (P-value<0.0001). Inhibition of biofilm formation at 500 µg.mL^-1^ concentration was 53.3%, 51.85%, and 45% for *S. aureus, A. baumannii*, and *P. aeruginosa* biofilms, respectively. At a 250 μg.mL^-1^ concentration, it had no inhibitory effect on *A. baumannii*, while it had about 53% inhibitory effect on *S. aureus* and about 30% on *P. aeruginosa*. The inhibitory effect of the peptide on these 3 bacterial species can be seen in Figure 3. Polymyxin B was tested as the positive control; however, it was unexpectedly ineffective in the inhibition of biofilm formation and metabolism, except for its effect on the biofilm metabolism of *S. aureus*.

By analyzing the results of biofilm metabolism, it was found that the highest peptide concentration, 1000 μg.mL^-1^, did not significantly decrease the biofilm of cells' metabolism, except on the *A. baumannii* (P-value < 0.01). After 20 h of incubation in the presence of TTC dye, the metabolism rates of S*. aureus, A. baumannii*, and *P. aeruginosa* biofilm were 70%, 68.75%, and 88%, respectively. Other concentrations did not also show any significant decrease in the rate of metabolism. It can be concluded that the peptide could not effectively eradicate the biofilm.

### 4.7. Structural Analysis of mCHTL131-140 Using Circular Dichroism

**Figure 4.**
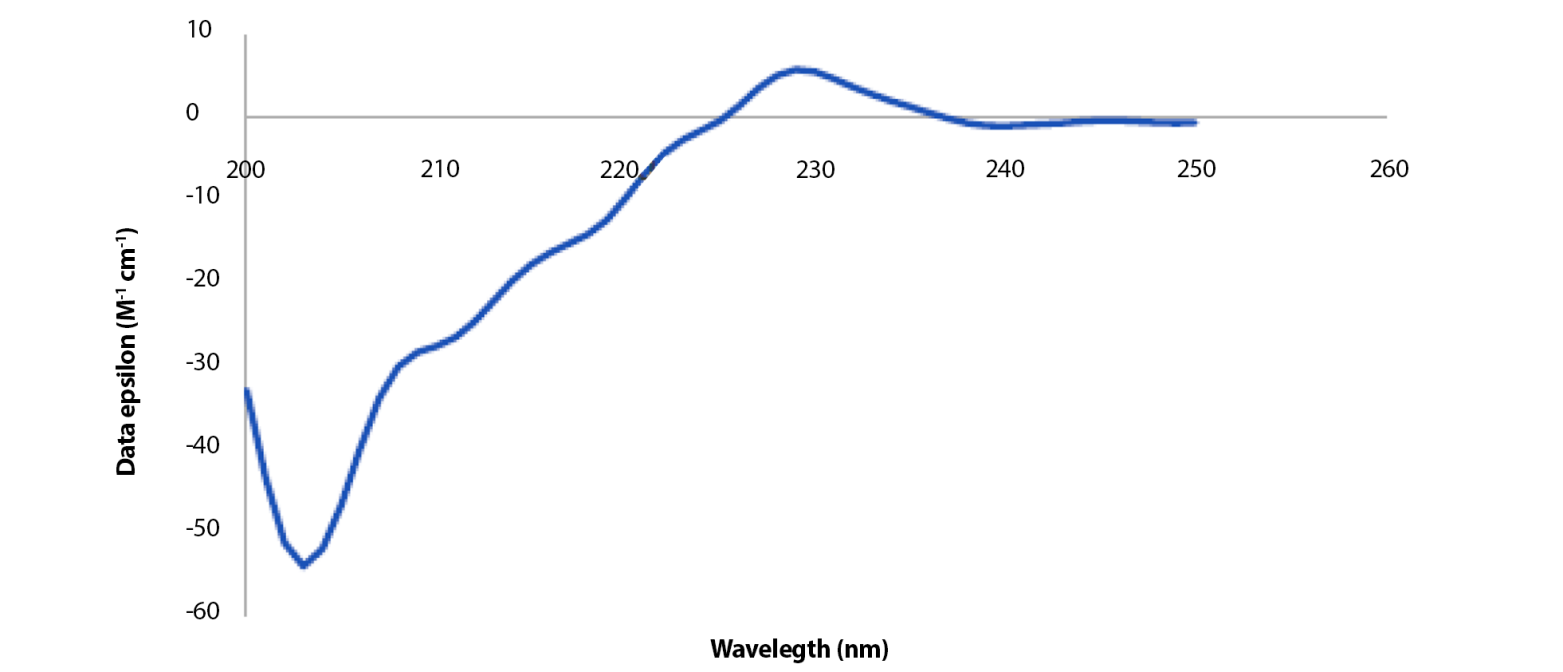
Circular dichroism (CD) spectrum of the designed peptide dissolved in 1X PBS; there is a very distinct negative peak around 200 nm and a clear positive peak about 230 nm.

The peptide was dissolved in 1X PBS solution in order to examine the structure. Analyses were conducted using the prepared solution at 1000 µg.mL^-1^ concentration. The presence of negative peaks at 222 nm and 208 nm and a positive peak at about 193 nm in the CD spectra are indicative of an alpha-helix structure. In contrast, negative peaks at 218 nm and a positive peak at 195 nm region characterize the structure of beta sheets in the CD spectrum. As shown in Figure 4, 2 peaks that are indicative of neither alpha helix nor beta-sheet structures can be seen: a very distinct negative peak at 200 nm and a clear positive peak at 230 nm. As a result, random coil configurations make up the majority of the peptide structure. The K2D3 online server (35), a free server for protein secondary structure estimation based on the CD spectrum, was employed. This server conducted the following analysis of the peptide structure based on the provided CD spectrum: 1.25 % α-helix, 28.5 % β-sheets, and 70.1% random coil. The CD spectrum results are comparable to the CFSSP server's prediction of the peptide's secondary structure (36). The secondary structure of the peptide was predicted using the CFSSP server before obtaining the CD spectrum; the outcomes of this prediction showed 25% α-helix, 41.6% β-sheets, 8.3% turn, and 25% random coil. It revealed that β-sheets were the most prevalent secondary structure, while the values for random coil and α-helix structures were equal. However, the CD spectrum contradicted this prediction and presented the random coil as the main structure, followed by the β-sheets structure.

### 4.8. mCHTL (131-140) Cytotoxicity Toward HDF Cells

**Figure 5.**
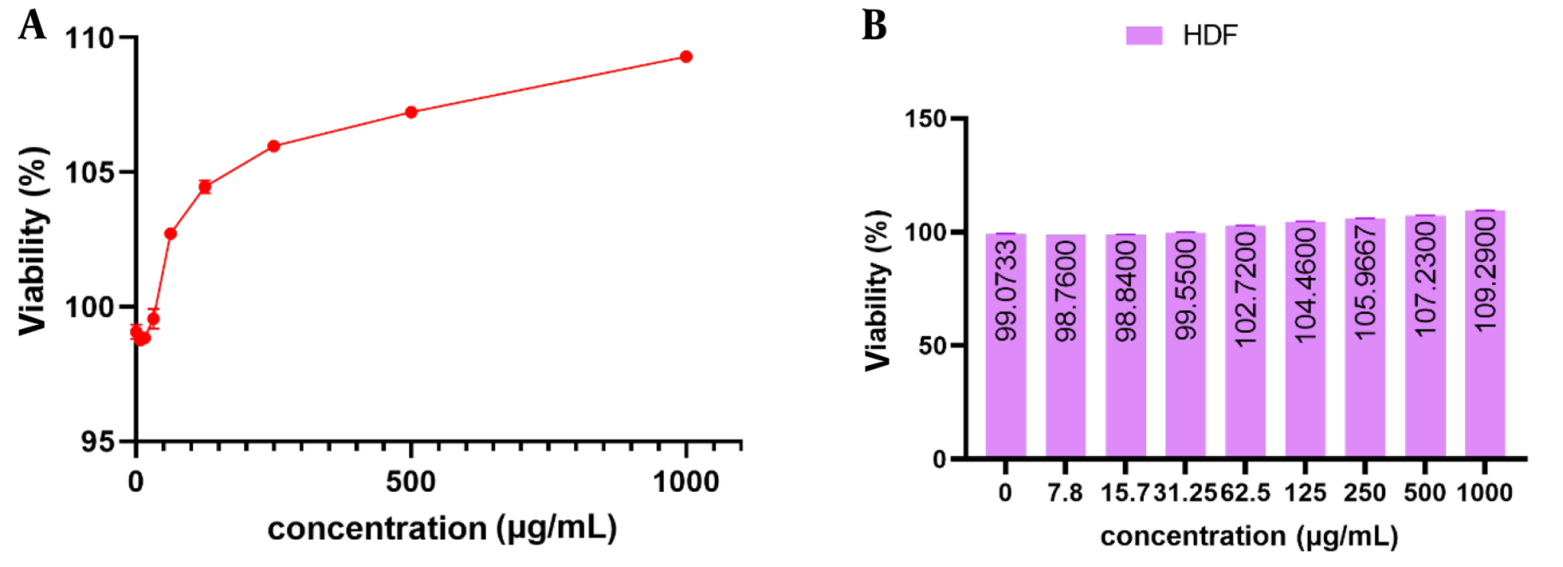
The viability percentage of human fibroblast (HDF) cells treated with different concentrations of mCHTL 131-140 as observed in two different forms of line (A) and column (B) charts. mCHTL131-140 had no toxic effect on HDF cells. There were no significant changes in cell viability compared to the control.

The experimental findings showed that mCHTL (131-140) did not exhibit any discernible toxicity toward the growth and proliferation of HDF cells. The findings are depicted in Figure 5. It is evident that in the positive control sample lacking the peptide, the growth rates of HDF cells after 24 hours of incubation were 99.073 % ± 0.271. At doses of 7.8, 15.7, and 31.25 μg.mL^-1^, the HDF cell proliferation rate was 98.76% ± 0.00, 98.84% ± 0.139, and 99.55% ± 0.367, respectively, extremely close to the control sample. At a concentration of 62.5 μg.mL^-1^, the cell exhibited a proliferation rate exceeding 100%. Subsequently, at a concentration of 1000 μg.mL^-1^, the cell proliferation rate was measured to be 109.29% ± 0.139.

## 5. Discussion

Rational ab initio design, redesigning and optimizing existing AMPs, de novo design, quantitative structure-activity relationship (QSAR) computational modeling, screening, and other computational techniques have all been utilized to help us design new AMPs. Nevertheless, only a few synthetic AMPs have attained clinical therapeutic benefits. Given that AMP discovery and in vitro analysis can be time-consuming and expensive, computational tools can be used to design and anticipate AMP's function through various AMP databases and different machine learning algorithms (such as ANN, SVMs, RF, discriminate analysis (DA), WEKA, and deep learning) (37).

In the present study, a rational design approach was used to design a novel multifunctional AMP. For this purpose, CTHL-2 was selected as the template protein because previous studies confirmed that this protein exhibits promising antimicrobial properties (30). This sequence was previously used as a template to identify peptide sequences with antimicrobial properties. Molhoek et al. showed that a short sequence derived from CTHL2 (F (2, 5, 12) W) had bactericidal activity and neutralizing effect on bacterial lipopolysaccharide. They reported that this peptide could inhibit *Staphylococcus epidermidis*. In another study by Molhoek et al., head-to-tail cyclization of F(2, 5, 12)W and substitution of I- to D- amino acids caused a dramatic increase in antibacterial activity and lipopolysaccharide neutralization effect (38, 39). Since some members of cathelicidins have the potential to heal wounds, the peptide motifs extracted from CTHL2 might also have wound-healing and anti-microbial activities.

In this study, the amino acid sequence from 131 to 140 on the CTHL2 was identified as a possible antibacterial peptide using the CAMP_R4_ server. Two amino acids of phenylalanine were replaced with tryptophan, and 2 amino acids of lysine were added to the sequence to improve the antimicrobial potential and reduce the hemolytic activity of the putative AMP. Bioinformatics study using freely available online servers predicted that RKWLRKIRRWRK was highly likely to have anti-bacterial, anti-biofilm, anti-fungal, and anti-viral properties.

Jiang et al. demonstrated how net charge and positively charged residues on the polar face considerably affect the antibacterial and hemolytic action of alpha-helical AMPs. They first selected V618—a peptide with antibacterial action. They declared that by lowering the net charge to (+4), the antibacterial and hemolytic actions became completely inert. In contrast, the analogs of the V618 were made more active in antibacterial activity with low-level hemolytic activity by systemically increasing the net charge from +4 to +8 (40). This newly designed peptide has a net charge of +8, which is expected to be a reasonably positive charge and provide strong antibacterial activity. Some studies have shown a direct relationship between the charge and hemolytic properties of the peptides (41). However, despite having 8 net positive charges, mCHTL131-140 did not show any hemolytic properties.

The mCHTL131-140 had the best performance against *A. baumannii* and *E. faecium* with the MIC of 7.8 µg.mL^-1^ and 62.5 µg.mL^-1^, respectively. In addition, the MIC against *P. aeruginosa* was 62.5 µg.mL^-1^. Since *A. baumannii* and *P. aeruginosa* are gram-negative bacteria, and *E. faecium* is a gram-positive bacterium, we can conclude that mCHTL131-140 would be active against both Gram-positive and Gram-negative bacteria. Nevertheless, its performance was even better on gram-negative bacteria.

*A. baumannii* is a gram-negative bacterial pathogen that has developed several resistant strains that have caused serious problems in treatment. It can cause a range of wound, skin, and urinary tract infections, as well as pneumonia and bacteremia. Multidrug-resistant (MDR) *Acinetobacter* may be resistant to one or more of the 3 classes of antibiotics, including penicillin and cephalosporin, aminoglycoside, and fluoroquinolones. Recently, carbapenem-resistant *A. baumannii* has ranked first in terms of warning for bacterial infection according to the World Health Organization's (WHO) list of 12 bacteria, indicating an urgent need to design new drugs (42).

Previously, Jakiewicz et al. examined how 8 well-known AMPs (CAMEL, LL-37, aurein 1.2, citropin 1.1, omiganan, pexiganan, temporin A, and r-omiganan) could kill *A. baumannii*, which is one of the most problematic pathogens to treat due to its proclivity to develop resistance and cause severe, difficult-to-treat illnesses. The outcomes showed that all peptides were highly active against the bacteria's planktonic forms; among them, Pexiganan and CAMEL had the highest potency. Citropin and LL-37 ranked second, with a lower level of activity (43). The MIC of mCHTL131-140 for *A. baumannii* (7.8 µg.mL^-1^) is comparable with that of LL-37 (16 µg.mL^-1^), one of the most famous natural cathelicidins. Furthermore, the peptide's MIC is comparable to that of another synthetic peptide (Octominin), which has an MIC of 5 µg.mL^-1^ for *A. baumannii* (44).

Similar to the target model protein in the current study, Dijk et al. looked for key components in the host defense peptide cathelicidin-2 (CATH-2) from chickens. To this end, hinge proline-altered analogs of CATH-2 and shortened peptides were created and evaluated for their antibacterial, immunomodulatory, and cytotoxic properties. They discovered that peptide C1-15, which corresponds to CATH-2's N-terminal helix and the hinge region, displayed the most growth inhibition against all the examined Gram-positive and Gram-negative bacteria (45). The mCHTL131-140 was extracted from a different part of the CATH-2 sequence compared to the peptide C1-15; however, it showed comparable antimicrobial features.

The findings revealed that mCHTL131-140 could also have anti-fungal activities against 2 of the most prevalent pathogenic fungi: *C. albicans* and *A. flavus*. Some studies focused on the in silico design of peptides that could show greater antifungal activities than the source sequence. For instance, the study by Ciociola et al. examined the structural and candidacidal capabilities of in silico-designed peptides (ISDPs), which were produced by changing the amino acids in the parent peptide KKVTMTCSAS. All of the ISDPs were able to treat the Candida infection model without harming mammalian cells, and they were more effective in vitro than the original peptide (46).

Moreover, although the in silico anti-biofilm activity prediction results were promising, the results of the experimental test on *P. aeruginosa* were not highly reasonable. The most effective anti-biofilm peptides available today have broad-spectrum activity against the biofilms created by the most dreaded antibiotic-resistant organisms and can eradicate biofilms at concentrations as low as 1 µg.mL^-1^ (47). Compared to the results reported, mCHTL131-140 did not have significant anti-biofilm activity (1000 μg.mL^-1^ compared to 1 μg.mL^-1^).

In terms of cytotoxicity to human cells, the results of hemolytic and toxicity tests against fibroblast cells showed that in the concentration range of antimicrobial activities, mCHTL131-140 did not cause lysis of red blood cells, and it also did not have a toxic effect on the growth of fibroblast cells. Consequently, it could be predicted that the designed AMP would not have toxic effects on all human cells. In fact, it was assumed that hemolysis could be employed as the primary index for the initial assessment of cellular toxicity; however, a study showed that the peptides with more hemolytic activity were less toxic against the HaCaT, HepG2, and HeLa cells. These researchers also proposed that the studied peptides would display similar toxicities against different cell types (41).

### 5.1. Conclusions

In conclusion, the capability to rapidly eliminate bacterial infections and accelerate wound healing makes antimicrobial peptides an excellent candidate for treating ulcers. However, their clinical applications are limited since many natural antimicrobial peptides exhibit cytotoxic activities. On the other hand, identifying and extracting bioactive peptides from natural resources is a laborious and low-yielded process. In this study, a peptide was designed through an in silico approach, which had anti-bacterial and anti-fungal properties and was not toxic to human red blood and human dermal fibroblast cells. These promising results confirmed that using bioinformatics methods makes it possible to reduce the time and effort and overcomes the disadvantages of experimental approaches in finding the bioactive peptides. We will be examining the in vitro and in vivo performance of the peptide for wound healing in future studies. If the anti-inflammatory and regenerative capabilities of this peptide are approved by further studies, it can be clinically applied with high potential for treating chronic wounds.

## Data Availability

Data will be available from the corresponding author upon request.
